# Supplementary material for: A new neornithischian dinosaur from the Upper Jurassic Tiaojishan Formation of northern China
Source: PeerJ. 2025 Jul 11;13:e19664. doi: 10.7717/peerj.19664 (PMC12258161; doi:10.7717/peerj.19664)
Supplement: Supplemental Information 4 — We carry out the ’common mapping of characters’ function on the the dataset ’unstable excluded’ in Supplemental Files to summarize the changes in morphological characters from early-diverging neornithischians to Ornithopoda. [file peerj-13-19664-s004.docx]

The overall trend of characteristic evolution from early-diverging neornithischian taxa to later-diverging Ornithopoda taxa includes these changes:

1.The number of the premaxillary teeth decreases and the premaxillary teeth even become absent;

2. The contact between the posterolateral process of the premaxilla and the lacrimal changes from absent to present, excluding the jugal from the antorbital fossa;

3. The ventral margin of the premaxilla gets deflected ventrally to maxillary tooth row and flares laterally;

4. The external naris extend posteriorly to lie above the maxilla and their ventral border rise;

5. The edentulous maxillary anterior margin length increases;

6. The antorbital becomes observable and from subtriangular to oval or subsquare,

7. The position of the antorbital changes from partly below the orbit to entirely anterior to the orbit;

8. The suture between the maxilla and the jugal changes from scarf joint to ‘finger-in-recess’ joint;

9. The ventral edge of the jugal curves more strongly posteroventrally;

10. The posterior process of the jugal changes from weakly expanded to either forked or strongly expanded and bluntly truncated and expands more towards the squamosal;

11. The postorbital projects more into the orbit;

12. The length of the squamosal process of the postorbital increases;

13. The free portion of the quadrate gets longer;

14. The quadrate condyle articular surface changes from ventromedially inclined to either ventrolaterally or horizonal,

15. The quadrate foramen changes from absent to present;

16. The frontal gets longer and narrower;

17. The depression on lateral surface on the squamosal continuous with the caudodorsal infratemporal fenestra extends more to above the quadrate head;

18. The contribution of supraoccipital to the dorsal margin of foramen magnum becomes less;

19. The dorsal margin length of the supraoccipital increases;

20. The ventral margin length of the supraoccipital decreases;

21. The relative width of the supraoccipital in the skull decreases;

22. The basal tuber of the basisphenoid gets thicker anteroposteriorly,

23. The orientation of the basipterygoid processes in the basisphenoid deflects more posteriorly;

24. The basipterygoid process articular facet for the pterygoid gets bigger and decreases in relative dorsal length;

25. The pterygoid mandibular process decreases in relative length;

26. The predentary gets longer and more pointed,

27. The oral margin of the predentary gets denticulate;

28. The ventral process of the predentary changes from single to bifurcated;

29. The diastema between the first dentary tooth and the predentary changes from absent to present;

30. The dentary length increases;

31. The number of dentary teeth increases from less than 15 to more than 20;

32. The number of the maxillary teeth increases from less than 15 to more than 20;

33. The shape of cheek teeth changes from subtriangular to diamond-shaped;

34. The shape of cheek tooth crown changes from triangular to rounded;

35. The apicobasally extending ridges on the labial surface of the cheek teeth changes from absent to present;

36. The apicobasally extending ridges on the labial surface of the cheek teeth changes from not confluent with denticles to confluent with denticles;

37. The primary ridge along cheek teeth gets more prominent;

38. The maxillary tooth ridge gets more prominent than the dentary one;

39. The crown height of cheek teeth increases;

40. The anteroposterior width of maxillary crowns become narrower than dentary crowns;

41. The shape of the marginal ornamentations changes from serrations to denticles;

42. The asymmetry of enamel distribution increased;

43. The level of the jaw joint becomes lower;

44. The number of cervical vertebrae increases from 9 to more than 10;

45. The length of cervical vertebrae decreases, their postzygapophyses get more arched;

46. The number of dorsal vertebrae increases from 12 or 13 to more than 16;

47. The height of dorsal vertebrae neural spine increases;

48. The number of sacral vertebrae increases from 2 to more than 6;

49. The length of anterior vertebrae transverse processes relative to neural spine height decreases;

50. Proximal caudal neural spines increase in height;

51.The arrangement of ossified tendons changes from longitudinally arranged to basket-like arrangement of fusiform tendons in caudal region;

52.The relative length of the humerus increases;

53.The shape of proximal carpals changes from ovoid to block-shaped;

54. The articulation between carpals and metacarpal I changes from free to co-ossified;

55. The proximal ends of metacarpals become block-like;

56. The relative length of metacarpal I and II decrease;

57. The shape of metacarpal I changes from elongated to block-like;

58. The shape of manual digit I ungual changes from claw-shaped to subconical;

59. The phalangeal number of manual digit III decreases from 4;

60. The relative length of manual digits II–IV decreases;

61. The ungual shape of manual digits II and III changes from claw-shaped to hoof-shaped;

62. The relative length of phalanx 1 of manual digit V changes increases;

63.The dorsal margin of iliac blade changes from convex midsection to horizonal;

64. The brevis and fossa change from facing ventrolaterally and visible in lateral view to facing ventrally and not visible in lateral view;

65. The eversion of dorsal margin of postacetabular process changes from absent or weak to prominent;

66. The relative length of postacetabular process decreases;

67. The supra-acetabular crest becomes absent;

68. The relative distance between the ischial process of the ischium and the pubic peduncle decreases;

69. The length of the pubis shaft decreases;

70. The anterior pubic blade expands;

71. The prepubic process changes from rod-like to either dorsoventrally or mediolaterally compressed;

72. The angle between the prepubic process and the pubic shaft increases;

73. The obturator foramen changes from a foramen to a notch;

74. The femoral shape in lateral view changes from bowed anteriorly along length to straight;

75. The relative femora length increases;

76. The anteroposterior length of the greater trochanter increases;

77. The femoral ligament sulcus becomes more prominent;

78. The anteroposterior length of the anterior trochanter decreases;

79. The anterior trochanter of the femur gets more proximally to the femoral head;

80. The fourth trochanter of the femur gets more distal to the head;

81. The anterior intercondylar groove on distal end of femur changes from absent to present;

82. The medial condyle on the posterior intercondylar groove of the femur gets inflated laterally;

83. The astragalar ascending process changes to subtriangular flange;

84. The medial distal tarsal changes from articulating metatarsals II and III to only metatarsal II;

85.The relative length of pedal phalanges decreases;

86. The pedal digit I goes absent.
